# Determinants of Non-emergency Use of Control Interventions in Older Canadian Psychiatric Inpatients: Analysizing the InterRAI Mental Health Electronic Health Records

**DOI:** 10.3389/fpsyt.2021.744341

**Published:** 2021-09-20

**Authors:** Gary Cheung, Tina M. Mah, Yoram Barak, John P. Hirdes

**Affiliations:** ^1^Department of Psychological Medicine, School of Medicine, University of Auckland, Auckland, New Zealand; ^2^Schlegel–University of Waterloo Research Institute for Aging, Waterloo, ON, Canada; ^3^School of Public Health Sciences, University of Waterloo, Waterloo, ON, Canada; ^4^Department of Psychological Medicine, School of Medicine, University of Otago, Dunedin, New Zealand

**Keywords:** control interventions, geriatric psychiatry, restraints, aggression, delirium

## Abstract

**Background:** The use of control interventions (CIs; acute control medications, physical/mechanical restraint) is associated with negative physical and psychological outcomes, particularly in older adults who are physically vulnerable. The aims of this study were to: (i) report the rates of CI use in older psychiatric inpatients (age 65 – 84 and age 85+), and compare them with younger age groups (18 – 44, age 45 – 64); and (ii) identify the factors associated with non-emergency CI use in older psychiatric inpatients.

**Methods:** Routinely collected interRAI Mental Health assessments from 2005 – 2018 in Ontario, Canada, were analyzed to determine the rates of CI use. Logistic regression models were used to examine the sociodemographic and clinical determinants of non-emergency and any CI use.

**Results:** There were 226,119 (female: 48.6%) interRAI assessments, and 85% of those assessed were under 65 years of age. The rates of non-emergency CI use in the four age groups were: 18 – 44 = 9.4%, 45 – 64 = 8.3%, 65 – 84 = 9.9%, 85+ = 13.2%. The most significant determinants of non-emergency CI use in older adults were highest impairments in activities of daily living (ADL Short Form score 8–16: OR = 2.72, 95% CI = 2.42 – 3.06), highest levels of aggression (Aggressive Behavior Scale score 4 – 6: OR = 1.76, 95% CI = 1.57 – 1.98), and highest levels of positive psychotic symptoms (Positive Symptoms Scale score 9+: OR = 1.65, 95% CI = 1.43 – 1.90). Delirium, cognitive disorder diagnosis, cognitive impairment, and falls were also associated with increased CI use odds, as were having the reasons for admission be danger to self, danger to others or inability to care for self. Females were less likely to have non-emergency CI use (OR = 0.84, 95% CI = 0.73 – 0.95). Patients admitted from long-term care homes had significantly greater odds of non-emergency CI use compared with community admissions (OR = 1.18; 95% CI = 1.07 – 1.29).

**Conclusion:** The higher rates of non-emergency CI use in older psychiatric inpatients is concerning. Alternative non-pharmacological and person-centered management strategies should be considered to support older psychiatric inpatients with functional impairment, positive symptoms, aggressive behavior, cognitive impairment and delirium. The use of CIs could be incorporated as a quality improvement activity to monitor changes at various service provision levels.

## Introduction

Control interventions (CIs) are often used as a last resort to maintain the safety of behaviorally disturbed psychiatric patients. These interventions include the use of acute control medications, mechanical restraints, chair that prevents rising, physical/manual restraint by staff and seclusion room. In addition to the management of aggression, physical restraints have been used in behaviorally disturbed older adults to carry out medical regimens and to prevent disruption of tubes and dressings, wandering and falls (1–3). Similar rationales for restraint use in a psychogeriatric inpatient unit that were previously reported include prevention of injury to patients, maintenance of treatment regimens, prevention of disturbance to other people, and protecting patients from harm (4). In psychiatry, reported risk factors most frequently associated with the use of coercive measures are male gender, young adult age, foreign ethnicity, schizophrenia, involuntary admission, aggression or trying to abscond and the presence of male staff (5). There is also evidence in the literature to suggest cognitive impairment and dementia are associated with restraint use in various clinical settings including nursing homes, medical wards and psychogeriatric inpatient units (3, 6–9).

The debate on balancing this form of control and possibly coercive treatment against patient autonomy is ongoing. A recent systematic review found the prevalence of restraint use with psychiatric inpatients was between 4 and 20% (5); whereas its prevalence ranged from 33 to 68% in general hospitals (10). While historical rates as high as 64% have been reported for nursing homes, the use of physical restraints and inappropriate use of antipsychotics has dropped precipitously in nursing homes as a result of widespread quality improvement (11, 12). National rates of physical restraint use in Canadian nursing homes are now below 5% (13). The rates of CI use vary a great deal between psychiatric units, regions and countries (14, 15). For example, the mean episodes of physical restraint in a 2-year period, adjusted for bed numbers and occupancy rates, were between 0 and 59.1% in five psychogeriatric admission wards in Victoria, Australia (12).

The use of CIs is associated with many negative physical and psychological outcomes, particularly in older adults who are physically vulnerable. These include falls, pressure injuries, depression, aggression, deep vein thrombosis/pulmonary embolism, longer length of hospital stay and death (2, 7, 16–18). A survey of Finnish psychiatric inpatients aged between 18 and 65 years found that patients were unsatisfied with their overall treatment following physical restraint or seclusion (19). In Canada, practice standards state that nurses should employ multi-dimensional and inter-disciplinary strategies to minimize use of restraints in all care settings (20).

Research on restraint practice in psychiatric services for older adults has received little attention (7). The interRAI Mental Health (interRAI MH) is a comprehensive standardized instrument for evaluating the needs, strengths and preferences of adults with mental illness in inpatient psychiatric settings (21). It provides an assessment of key domains of function, mental and physical health, substance use/behaviors, social support and service use. This present study used a large dataset of routinely collected interRAI MH assessments from 2005 – 2018 in Ontario, Canada. Although falls risk is considered to be one of the main risk factors associated with the use of physical and mechanical restraint in nursing homes, hospitals, and home care (2), none of the 49 studies included in a recent systematic review of restraint use in psychiatry included falls as a risk factor in their investigation (5). The use of interRAI MH in our study allowed an investigation of sociodemographic, physical, falls, cognitive, functional, and psychosocial factors associated with the use of CIs.

The aims of this study were to: (i) report the rates of emergency and non-emergency use of CIs in older psychiatric inpatients (age 65 – 84 and age 85+) and compare them with younger age groups (18 – 44, age 45 – 64); and (ii) identify factors associated with non-emergency use of CIs in psychiatric inpatients aged 65 and over. The emergency use of CIs could be justified to prevent harms in acute or emergency clinical situations, however, non-emergency use of CIs warrants further examination. A better understanding of the determinants of non-emergency use of CIs could potentially identify people who are at risk of being subjected to unnecessary coercive measures. Emergency and non-emergency CI use are differentiated in care planning guidelines associated with interRAI's suite of mental health instruments; (22, 23) and restraint use can be benchmarked as a mental health quality indicator using these instruments (24).

## Methods

### Setting and Participants

The study sample includes psychiatric inpatients (aged 18+) with completed interRAI MH admission assessments^1^ between the last quarter of 2005 and the first quarter of 2018 in Ontario, Canada. The study sample was stratified into four age groups: age 18 – 44, age 45 – 64, age 65 – 84 and age 85+. Beginning in 2005, an interRAI MH assessment is routinely required for each patient within 72 h of admission, at discharge and every 90 days for longer stays by all Ontario psychiatric inpatient hospitals and units. There were 82,411 discharges from 74 psychiatric hospitals and psychiatry units located in general hospitals in Ontario in 2018 (25).

Ethics approval was obtained through the Office of Research at the University of Waterloo (ORE # 30372 and #15436).

The interRAI MH assessment is designed for use by mental health professionals such as nurses, social workers, psychiatrists, psychologists and occupational therapists (21). The assessment process involves clinical consideration of multiple sources of data including communication with the person, the primary support person and other members of the clinical team, observation of the person, review of medical records and other relevant documents (21, 22, 26, 27). All items include standardized definitions, statements of intent, coding guidelines, and illustrative examples to be used by assessors. There are 21 sections in the interRAI MH Assessment Form which includes the domains of intake and initial history, mental state indicators, substance use, harm to self and others, behavior, cognition, functional status, communication, and vision, health conditions, stress and trauma, medications, service utilization and treatments, nutritional status, social relations, employment, education, and finances.

#### Outcome and Independent Variables

A number of clinical outcome scales and Clinical Assessment Protocols (CAPs) are embedded in the interRAI MH assessment (28). CAPs are used to identify specific clinical conditions or situations to help and inform care plans. In emergency situations, the CI CAP is triggered when a person who (i) has experienced a physical restraint (mechanical, chair prevents rising, or physical or manual restraint by staff), seclusion or acute control medications in the 3 days prior to the assessment; and (ii) was in a psychiatric emergency situation, as indicated by one or more of the following: suicide attempt in the 3 days prior to the assessment, violence toward others in the 3 days prior to the assessment, score of 13 or higher on the long version of the interRAI Positive Symptoms Scale, extreme behavior disturbance in the 7 days prior to the assessment, command hallucinations in the 3 days prior to the assessment, and Aggressive Behavior Scale score of six or higher (28). In non-emergency situations, the CI CAP is triggered when a person who has experienced restraints, seclusions, or acute control medications use in the 3 days prior to the assessment but were not in a psychiatric emergency situation as described above, or has had a long-term history of ongoing restraint use, perhaps in another care setting (28).

The following independent variables and scales listed below were chosen based on the existing literature on CI summarized elsewhere (29).

##### Clinical and Sociodemographic Variables

Age, gender, ethnicity, marital status, reasons for admission, admission source, legal admission status, capacity to consent to treatment, legal guardian/substitute decision-maker, Diagnostic and Statistical Manual of Mental Disorders-IV diagnosis and past mental health admission.

##### Clinical Outcome Scales and CAPs

A summary of available CAPs and scales for the interRAI mental health instruments is provided elsewhere (22, 24). Those considered in the current study include: Activities of Daily Living (ADL) Short Form, Aggressive Behavior Scale, Risk of Harm to Others Scale, Severity of Self-harm, Self-care Index, Cognitive Performance Scale, Depressive Severity Index, Positive Symptoms Scale, Falls CAP (28).

*ADL Short Form –* provides a summary measure of the person's ability to perform ADLs based on four items: personal hygiene, toilet use, locomotion and eating. The scale has a range of 0 – 16, with higher values indicating greater difficulty in performing activities.

*Aggressive Behavior Scale* – measures the frequency and diversity of aggressive behaviors including verbal abuse, physical abuse, socially inappropriate behavior, disruptive behavior, and resisting care. Scores range from 0 to 12, with higher scores indicating greater frequency and diversity of aggressive behavior (0 = no signs of aggression; 1 – 4 = mild to moderate aggression; 5 += more severe aggression).

*Risk of Harm to Others* – reflects risk of harm to others with scores range from 0 to 6 (higher scores indicate increased risk of harm to others).

*Severity of Self-harm* – reflects risk of harm to self with scores range from 0 to 6 (higher scores indicate increased risk to self-harm).

*Self-care Index* – reflects risk of inability to care for self due to psychiatric symptoms with scores range from 0 to 6 (higher scores indicate decreased ability to care for self).

*Cognitive Performance Scale* – describes the person's cognitive status and scores ranged from 0 to 6 (0 = intact; one = borderline intact; two = mild impairment; three = moderate impairment; four = moderate to severe impairment; five = severe impairment, six = very severe impairment).

*Depressive Severity Index* – is a measure for depressive symptoms with higher scores indicating more depressive symptoms (scores range from 0 to 15).

*Positive Symptoms Scale (Long Form)* – measures the frequency of positive psychotic symptoms such as hallucinations, delusions, abnormal thought process, inflated self-worth, hyperarousal, pressured speech and abnormal/unusual movements. Scores range from 0 to 24 with higher scores indicating higher levels of positive symptoms.

*Falls CAP* – is triggered when the person has had one of more prior falls.

### Statistical Analysis

Data were analyzed using the Statistical Analysis Service (SAS) 9.4 software. Descriptive statistics for clinical and sociodemographic variables were obtained for the four age groups. Bivariate analysis with chi-squared tests was used to investigate the significance of the relationships between independent clinical and sociodemographic variables and non-emergency CI use (dependent variable) in older psychiatric inpatients (age 65+). All independent variables that proved to be statistically significantly (*p* < 0.05) in the bivariate analysis were entered into logistic regression models predicting non-emergency use of CIs as well as any of CIs. We also included assessment year as a covariate in the models. Results are presented as odds ratios (OR, with 95% confidence intervals, CI) which should be interpreted as the effect that an independent variable has on the odds of CI use. C-statistics were used to provide information on the explanatory power of the models.

As described earlier, the non-emergency situation CI CAP is triggered when a person was not in a psychiatric emergency or has had a long-term history of restraint use in another care setting. The Aggressive Behavior Scale (score ≤5) and Positive Symptoms Scale (score ≤12) are considered in the definitions of non-psychiatric emergency situations being in the lower range of the scales. However, we included these two scales as independent variables because there may still be predictive value using these scales despite their distributions being attenuated based on the CAP coding rules.

## Results

A total of 226,119 unique adults aged 18+ were assessed at admission with the interRAI MH during the study period, and about 85% of those assessed were under 65 years of age (*n* = 191,402 under 65 compared with 34,717 aged 65 years or more). About half the sample (48.6%) was female (*n* = 109,981). Table 1 provides a profile of the use of CIs, clinical attributes, and sociodemographic characteristics of the sample divided into four age groupings (young adults aged 18 – 44: *n* = 114,976; middle-aged adults aged 45 – 64: *n* = 76,426; older adults aged 65 – 84: *n* = 30,138; oldest-old adults aged 85+: *n* = 4,579).

**Table 1:** Comparison of the use of control interventions, clinical and sociodemographic profiles of psychiatric inpatients across 4 age groups (18–44 years, 45–64 years, 65–84 years, 85+ years).

	**18–44 years**	**45–64 years**	**65–84 years**	**85+ years**
	***N* = 11,4976**	***N* = 76,426**	***N* = 30,138**	***N* = 4,579**
**Control Interventions CAP**				
Triggered (emergency situation)	9,646 (8.4)	4,888 (6.4)	3,071 (10.2)	691 (15.1)
Triggered (non-emergency situation)	10,807 (9.4)	6,352 (8.3)	2,986 (9.9)	603 (13.2)
**Types of control interventions**				
Acute control medications	16,423 (14.3)	8,884 (11.6)	3,961 (13.1)	751 (16.4)
Mechanical restraint	3,149 (2.7)	1,528 (2.0)	1,711 (5.7)	453 (9.9)
Chair prevents rising	146 (0.1)	379 (0.5)	1,608 (5.3)	509 (11.1)
Physical/manual restraint by staff	2,561 (2.2)	1,205 (1.6)	1,191 (4.0)	292 (6.4)
Seclusion room	5,683 (4.9)	2,677 (3.5)	947 (3.1)	127 (2.8)
Gender (Female)	52,004 (45.3)	38,739 (50.7)	16,547 (54.9)	2,691 (58.8)
**Marital status**				
Married	24,439 (21.3)	30,077 (39.4)	13,737 (45.6)	1,625 (35.5)
Never married	79,552 (69.2)	24,110 (31.6)	4,660 (15.5)	384 (8.4)
**Reasons for admission**				
Threat or danger to self	59,718 (51.9)	36,028 (47.1)	11,631 (38.6)	1,703 (37.2)
Threat or danger to others	21,146 (18.4)	10,595 (13.9)	6,947 (23.1)	1,457 (31.8)
Inability to care for self	35,652 (31.0)	27,305 (35.7)	16,253 (53.9)	2,738 (59.8)
Admission from long term care facility	1,225 (1.1)	2,308 (3.0)	5,439 (18.1)	1,715 (37.5)
**Inpatient status at time of assessment**				
Application for assessment	17,235 (18.8)	10,852 (18.0)	3,722 (16.1)	529 (15.6)
Voluntary	44,464 (48.5)	32,668 (54.1)	11,458 (49.6)	1,566 (46.1)
Informal	633 (0.7)	509 (0.8)	1,272 (5.5)	353 (10.4)
Involuntary	26,790 (29.2)	15,386 (25.5)	6,506 (28.1)	946 (27.9)
Forensic	2,534 (2.8)	1,010 (1.7)	167 (0.7)	2 (0.1)
Incapable of consenting to treatment	7,161 (6.2)	5,549 (7.3)	6,808 (22.6)	1,713 (37.4)
Has legal guardian/substitute decision-maker	5,926 (5.2)	5,112 (6.7)	8,052 (26.7)	2,124 (46.4)
**DSM-IV Diagnosis**				
Cognitive disorder	943 (0.8)	2,968 (3.9)	10,735 (35.6)	2,955 (64.5)
Delirium	27,334 (23.8)	19,164 (25.1)	12,458 (41.3)	2,334 (51.0)
Mood disorder	58,005 (50.5)	43,861 (57.4)	15,624 (51.8)	1,829 (40.0)
Schizophrenia	35,239 (30.7)	20,640 (27.0)	6,223 (20.7)	556 (12.1)
Substance use disorder	38,316 (33.3)	20,216 (26.5)	2,775 (9.2)	91 (2.0)
Personality disorder	12,652 (11.0)	5,584 (7.3)	1,012 (3.4)	85 (1.9)
Anxiety disorder	19,040 (16.6)	12,009 (15.7)	3,207 (10.6)	331 (7.2)
Intellectual disability	4,731 (4.1)	2,554 (3.3)	864 (2.9)	143 (1.7)
No past mental health admission	47,034 (40.9)	26,741 (35.0)	13,521 (44.9)	3,028 (66.1)
**Aggressive Behavior Scale**				
0	88,600 (77.1)	60,497 (79.2)	19,505 (64.7)	2,434 (53.2)
1–3	15,015 (13.1)	9,203 (12.0)	5,241 (17.4)	939 (20.5)
4–6	7,778 (6.8)	4,577 (6.0)	3,047 (10.1)	637 (13.9)
7–9	2,887 (2.5)	1,655 (2.2)	1,602 (5.3)	361 (7.9)
10–12	696 (0.6)	494 (0.7)	743 (2.5)	208 (4.5)
**Risk of Harm to Others Scale**				
0	32,899 (28.6)	24,418 (32.0)	9,424 (31.3)	1,317 (28.8)
1–2	51,590 (44.9)	35,324 (46.2)	11,580 (38.4)	1,488 (32.5)
3–4	17,044 (14.8)	10,034 (13.1)	4,340 (14.4)	720 (15.7)
5–6	13,443 (11.7)	6,650 (8.7)	4,794 (15.9)	1,054 (23.0)
**Severity of Self-harm**				
0	26,752 (23.3)	18,704 (24.5)	5,112 (17.0)	366 (8.0)
1–2	35,460 (30.8)	25,728 (33.7)	15,767 (52.3)	2,953 (64.5)
3–4	21,702 (18.9)	12,423 (16.3)	5,067 (16.8)	821 (17.9)
5–6	31,062 (27.0)	19,571 (25.6)	4,192 (13.9)	439 (9.6)
**Self-care Index**				
0	40,597 (35.3)	24,918 (32.6)	5,047 (16.8)	379 (8.3)
1–2	51,055 (44.4)	33,519 (43.9)	13,675 (45.4)	2,109 (46.1)
3–4	14,494 (12.6)	11,529 (15.1)	7,077 (23.5)	1,334 (29.1)
5–6	8,830 (7.7)	6,460 (8.5)	4,339 (14.4)	757 (16.5)
**Falls CAP**				
Triggered	4,632 (4.0)	5,891 (7.7)	4,927 (16.4)	1,120 (24.5)
**Cognitive performance scale**				
0	87,011 (75.7)	52,271 (68.4)	11,011 (36.5)	767 (16.8)
1–2	23,803 (20.7)	19,152 (25.1)	10,434 (34.6)	1,517 (33.1)
3–6	4,162 (3.6)	5,003 (6.6)	8,693 (28.8)	2,295 (50.1)
**Depressive Severity Index**				
0	29,239 (25.4)	17,828 (23.3)	8,327 (27.6)	1,386 (30.3)
1–3	36,090 (31.4)	23,827 (31.2)	10,723 (35.6)	1,667 (36.4)
4–7	30,128 (26.2)	20,614 (27.0)	6,724 (22.3)	948 (20.7)
8–15	19,590 (17.0)	14,157 (18.5)	4,364 (14.5)	578 (12.6)
**Positive Symptoms Scale**				
0	55,094 (47.9)	38,156 (49.9)	13,041 (43.3)	1950 (42.6)
1–3	22,651 (19.7)	15,165 (19.8)	7,243 (24.0)	1238 (27.0)
4–8	22,747 (19.8)	14,406 (18.9)	6,526 (21.7)	963 (21.0)
9–24	14,484 (12.6)	8,699 (11.4)	3,328 (11.0)	428 (9.4)
**ADL Short Form**				
0	107,452 (93.5)	67,410 (88.2)	17,265 (57.3)	1,413 (30.9)
1–2	4,723 (4.1)	4,569 (6.0)	3,859 (12.8)	666 (14.5)
3–4	1,613 (1.4)	1,939 (2.5)	2,344 (7.8)	515 (11.3)
5–7	599 (0.5)	977 (1.3)	2,245 (7.5)	577 (12.6)
8–16	569 (0.5)	1,531 (2.0)	4,425 (14.7)	1,408 (17.8)

The rates of CI use showed a slight curvilinear pattern in the four age groups with rates dropping off slightly from young adults to middle-aged adults, followed by steady increments of use in the two oldest adult groups. Rates were more than doubled from a low of 6.4% in middle-aged adults to a high of 15.1% in the oldest-old for emergency use of CI. Similarly, the rates of non-emergency use of CIs were lowest in the middle-aged group (8.3%) and highest in the oldest-old (13.2%).

Acute control medications were the most commonly used CI in all age groups, and they followed the abovementioned trend in age differences. Use of seclusion rooms was most prevalent among young adults; however, mechanical restraints, chairs that prevent rising, and physical/manual restraint by staff were all most likely used with the oldest-old inpatients.

Compared with other age groups, the oldest-old were most likely to be female, admitted due to inability of care for self or danger to others, admitted from a long term care facility, incapable of providing consent, have a legal guardian/substitute decision-maker. However, they were also most likely to not have had a prior mental health admission.

In terms of clinical characteristics, the older age categories were associated with more severe levels of risk of harm to others, aggressive behavior, problems with self-care, falls, cognitive impairment, and ADL impairment. On the other hand, older age groups tended to have lower or comparable scores for self-harm, depressive symptoms and positive psychotic symptoms compared with younger age groups.

With respect to provisional psychiatric diagnoses at admission, older adults and the oldest-old adults tended to have higher rates of cognitive disorders and delirium compared with younger and middle-aged adults. The converse was true for mood disorders, schizophrenia, substance use disorder, personality disorder, anxiety, and intellectual disability.

Table 2 shows the results of the logistic regression analysis examining the sociodemographic and clinical determinants of non-emergency CI use and any CI use. Having established that CI use was higher in older persons compared with young and middle-aged adults in Table 1, we narrowed our multivariate analyses to the subset of inpatients aged 65 and older (*n* = 34,716 for any CI use and *n* = 29,646 for non-emergency CI use vs. no CI use). The models showed good overall performance with a c-statistics of 0.78 and 0.83 for non-emergency and any CI use, respectively.

**Table 2:** Logistic regression models examining the sociodemographic and clinical determinants of any use of control interventions (CIs), and use in non-emergency situations only.

**Variables**	**Non-emergency CI use**	***p* value**	**Any CI use**	***p* value**
	***N* = 29,646**		***N* = 34,716**	
	**Odds Ratio**		**Odds Ratio**	
	**(95% CI)**		**(95% CI)**	
Age 85+ (ref = 65 – 84)	0.96 (0.86 – 1.06)	0.40	0.95 (0.87 – 1.04)	0.25
Female gender (ref = male)	0.83 (0.77 – 0.90)	<0.0001	0.79 (0.74 – 0.84)	<0.0001
Admitted from Long Term Care (ref = no)	1.19 (1.08 – 1.31)	0.0003	1.15 (1.07 – 1.24)	0.0002
Reasons for admission (ref = no)				
Threat or danger to self	1.18 (1.09 – 1.28)	<0.0001	1.24 (1.17 – 1.32)	<0.0001
Threat or danger to others	1.19 (1.08 – 1.31)	0.0003	1.35 (1.26 – 1.45)	<0.0001
Inability to care for self	1.40 (1.28 – 1.52)	<0.0001	1.26 (1.18 – 1.33)	<0.0001
Cognitive Disorder diagnosis (ref = no)	1.40 (1.28 – 1.53)	<0.0001	1.35 (1.25 – 1.46)	<0.0001
Any delirium indicators present (ref = no)	1.41 (1.29 – 1.54)	<0.0001	1.43 (1.33 – 1.54)	<0.0001
Falls CAP triggered	1.21 (1.10 – 1.33)	<0.0001	1.18 (1.10 – 1.28)	<0.0001
Aggressive Behavior Scale (ref = 0)				
1 – 3	1.50 (1.37 – 1.65)	<0.0001	1.67 (1.54 – 1.81)	<0.0001
4 – 6	1.77 (1.57 – 1.99)		3.40 (3.10 – 3.73)	
7 – 9	NA^a^		5.58 (4.95 – 6.29)	
10+	Na^a^		8.74 (7.31 – 10.46)	
Cognitive Performance Scale (ref = 0)				
1 – 2	1.14 (1.02 – 1.28)	<0.0001	1.14 (1.04 – 1.26)	<0.0001
3 – 6	1.50 (1.31 – 1.72)		1.54 (1.38 – 1.73)	
Positive Symptoms Scale (ref = 0)				
1 – 3	1.24 (1.13 – 1.37)		1.27 (1.17 – 1.38)	
4 – 8	1.51 (1.36 – 1.67)		1.62 (1.49 – 1.76)	
9+	1.74 (1.51 – 2.00)	<0.0001	2.07 (1.87 – 2.29)	<0.0001
ADL Short Form				
1 – 2	0.97 (0.86 – 1.20)	<0.0001	1.00 (0.90 – 1.10)	<0.0001
3 – 4	1.04 (0.90 – 1.20)		1.10 (0.98 – 1.23)	
5 – 7	1.22 (1.06 – 1.41)		1.24 (1.11 – 1.40)	
8 – 16	2.66 (2.37 – 3.00)		2.38 (2.15 – 2.62)	
Year (ref = 2005)^b^				
2006	0.80 (0.59 – 1.09)	<0.0001	0.87 (0.67 – 1.12)	<0.0001
2007	0.74 (0.54 – 1.00)		0.73 (0.56 – 0.95)	
2008	0.72 (0.53 – 0.98)		0.77 (0.59 – 1.00)	
2009	0.74 (0.54 – 1.00)		0.76 (0.59 – 0.99)	
2010	0.77 (0.57 – 1.05)		0.78 (0.60 – 1.01)	
2011	0.73 (0.54 – 0.99)		0.70 (0.54 – 0.91)	
2012	0.56 (0.41 – 0.77)		0.61 (0.47 – 0.80)	
2013	0.52 (0.38 – 0.72)		0.55 (0.42 – 0.72)	
2014	0.46 (0.34 – 0.63)		0.50 (0.38 – 0.65)	
2015	0.54 (0.40 – 0.73)		0.54 (0.42 – 0.71)	
2016	0.48 (0.35 – 0.65)		0.52 (0.40 – 0.68)	
2017	0.43 (0.31 – 0.58)		0.45 (0.35 – 0.58)	
2018	0.43 (0.30 – 0.62)		0.45 (0.34 – 0.61)	
Likelihood ratio chi-square	1261.2	<0.0001	8987.1	<0.0001
C statistic	0.76		0.83	

a *Non-emergency CAP trigger level excludes persons with Aggressive Behavior Scale ≥6 or Positive Symptoms Scale ≥ 13*.

b *2005 and 2018 include the last and first calendar quarters, respectively*.

The specification of the final models was based on the variables found to be significant for non-emergency use. That set of covariates was then applied to the model for any CI use to determine whether the covariates were differentially important for the two patterns of use.

Non-emergency CI use among older persons in psychiatry was affected by a combination of person-level factors as well as health system considerations. Function, cognition, and behavior were important clinical attributes associated with greater odds of CI use. For example, the odds were highest for the highest impairments in activities of daily living (ADL Short Form score 8 – 16: OR = 2.72, 95% CI = 2.42 – 3.06), highest levels of aggression (Aggressive Behavior Scale score 4 – 6: OR = 1.76, 95% CI = 1.57 – 1.98), and highest levels of positive psychotic symptoms (Positive Symptoms Scale score 9+: OR = 1.65, 95% CI = 1.43 – 1.90). Delirium, cognitive disorder diagnosis, cognitive impairment, and falls were also associated with increased odds, as were having the reasons for admission be danger to self, danger to others or inability to care for self. Females were less likely to have non-emergency CI use (OR = 0.84, 95% CI = 0.73 – 0.95).

However, after adjusting for all of these person-level clinical and demographic variables, there was an independent health-system effect related to source of admission. Those admitted from long-term care homes had significantly greater odds of non-emergency CI use compared with community admissions (OR = 1.18; 95% CI = 1.07 – 1.29) after controlling for reason for admission, cognition, ADL impairment, falls, behavior, positive symptoms, and delirium. We also found a historical trend that was statistically significant as of 2011, using 2005 as a reference point. This time period does not correspond to any change in legal framework of non-emergency use of CI; however, it does correspond with the launch of the Mental Health and Addictions Quality Initiative (MHAQI) (30). MHAQI was founded as a collaborative network among hospitals with inpatient psychiatric beds with the aims of pursuing joint quality improvement initiatives. Restraint use was an important initial focus of this network.

When the same covariates were applied to a logistic regression model for *any* CI use, there were few substantively important changes in the magnitudes of these associations. None of the odds ratios became non-significant and none changed direction in their relationships with CI use.

## Discussion

Although the association of age and CI use was reported as mixed in the literature (5, 7, 9), we found a clear trend of increasing CI use with age in this large Canadian psychiatric inpatient sample. Whether considering non-emergency use or any use of CIs, this approach to care was most common in older adults with a peak among the oldest-old. Previous research has not investigated the association between falls and the use of CIs in psychiatric settings (5), but our findings point to this as an important relationship. We found that clinical characteristics of older inpatients affected the use of non-emergency CIs, especially impaired ADLs, cognition, aggression and delirium. However, the use of CIs was also affected by where the person was admitted from after controlling for clinical, demographic, and diagnostic covariates.

The association of impaired ADLs and CI use found in this study is consistent with other international studies. A nationwide survey of institutions for older adults in Norway found that force or pressure was the most frequently used restraint method when performing activities of daily living of their residents (31). Inability to perform ADL activities was also found to increase the frequency of restraint use in a study of psychogeriatric inpatients in Germany (9). Impaired ADL in older adults can be associated with cognitive impairment, and practical guidance on the use of physical restraints with people with Alzheimer's disease has emphasized the consideration of the perceived benefits and potential harms of CI use, regular review of continued restraint use, limiting the use of restraint to a minimal level, and educating clinicians about the risks of physical restraint and safe practice when restraining a person (32).

It is particularly concerning to find that delirium was associated with non-emergency CI use in this analysis. It has been shown that physical restraints can lead to delirium, therefore, should not be used for patients at risk of delirium or have already developed delirium (33). The management of delirium requires addressing both the medical and psychiatric care needs of the patient. A previous study found that patients with delirium admitted directly to a collocated geriatric and psychogeriatric unit, where nurses were dually qualified in medical and psychiatric conditions, had better outcomes and shorter lengths of stay than patients who were transferred to other wards in the hospital (34). Other key components in preventing and managing delirium include staff education, systematic screening, multidisciplinary approach and a focus on non-pharmacological interventions (33). Antipsychotics are often used as an acute control medication in hyperactive delirium. This class of medication can sometimes be effective in treating aggression and agitation in older people with dementia; however, they are not without side effects and could potentially worsen the clinical course of delirium. Interestingly, a previous study found nursing home residents taking antidepressant medication had lower level of aggression (3). It was highlighted that aggression in nursing home residents could be a manifestation of untreated agitated depression. In the same study, pain and other common geriatric physical problems such as constipation and urinary tract infection could trigger aggression in older people with cognitive impairment.

The use of alternatives to physical restraints from the perspectives of older people and staff requires further research (35). Canadian long-term care homes have undergone a large scale reduction in restraint use over the last two decades demonstrating that such a change is entirely feasible (36, 37).

A Canadian qualitative study explored the views of family members of older people in long-term care facilities regarding alternatives to physical restraints and seclusion (38). Family members believed the need for restraint and seclusion could be reduced by creating a stimulating environment in the care facility, introducing individualized occupational therapy programs along with listening, communicating, and assessing the needs of the older people. Patients often thought their opinions were not included in their treatment planning (16). Staff working in acute old age psychiatry inpatient units in Australia thought aggressive behavior in their patients was related to the environment and aggression occurred because staff did not listen to patients (39). Another Australian study found nurses working in acute old age psychiatry inpatient units felt that there were no effective alternatives to the use of physical restraints and seclusion (40), a similar finding reported in a Hong Kong study (4).

An increase in staffing does not necessarily translate to a lower rate of restraint use (31). The reverse also being found that high workload and low percentage of registered nurses was not associated with greater restraint use in a study of 15 Dutch psychogeriatric nursing home wards (6). Restraint reduction programs can be effective in reducing the rates of physical restraint use. A meta-analysis of nine randomized controlled trials (RCTs)/cluster RCTs found significant effects with restraint reduction programs (41). These programs typically used education to improve the care provided to older people by helping carers to identify alternatives to restraint use and by providing information about the care of older people with dementia (42). Other interventions included providing a change-agent or an expert for ongoing consultations (41). For example, a restraint reduction program in a convalescent medical ward in Hong Kong resulted in the rates of restraint use reduced from 13.3 to 4.1% (8). Assessing communication and baseline behaviors could prevent CI use in people with dementia, in particular those behaviors that place a patient at risk of CI use, for example, falls risk, interference with treatment devices such as feeding tubes, intravenous lines, urinary catheter (43). In addition, appropriate education and support has been recommended to address the ethical and workplace cultural issues associated with the practices of restraint and seclusion (40).

There are four main applications in the use of interRAI instruments: care planning, outcome measurement, resource allocation and quality improvement (22). When the non-emergency CI CAP is triggered, appropriate person-level intervention to address the associated factors found in this study should follow as part of the care planning. The use of CIs, particularly in non-emergency situations, can be used as a quality indicator for performance monitoring at service/facility and population levels. Multimodal interventions involving leadership, policies, staff training and education are shown to reduce physical restraint use in inpatient psychiatric settings (44). There have also been quality improvement initiatives that effectively reduce physical restraint use in hospital settings (45, 46).

Quality of care should not be considered within health sectors alone. A more person-centered approach is to employ a health systems perspective to examine how individuals are cared for in different settings. The finding that prior long-term care placement was an independent predictor of non-emergency and any CI use, while controlling for numerous other covariates, is of great concern. These results raise the possibility of care driven not by personal needs, but by system-level factors that should be irrelevant to care strategies. This then begs the question of whether the care of persons who are transferred from long-term care to inpatient psychiatry facilitates received improved or worsened care for their mental health needs.

The main limitation of this study is that different types of CIs are collectively analyzed as one category of interventions. The determinants of each individual CI could be different. For example, Mah et al. found 72% of an earlier cohort of Ontario psychiatry inpatients restrained with a chair that prevents rising were 65 years and older (14). Chair restraint was the most frequently CI used in older inpatients, followed by mechanical/physical restraint and acute control medication (14). Future studies could examine the determinants of each type of CIs separately. Nevertheless, this study provides evidence about CI use rates that can serve as a baseline and monitored over time as a quality indicator at a population level. It also serves as a first step in highlighting the higher rates and the factors associated with CI use in older psychiatric inpatients. Indeed, immediate action should be taken to publicly report on the use of CIs in inpatient psychiatry in Canada, as is already done in the long-term care sector through the Canadian Institute for Health Information's public reporting portal (13).

## Conclusion

This study found higher rates of CI use in older psychiatric inpatients who are the most vulnerable group in our society. Non-emergency use of CIs in inpatient psychiatric units was associated with older people who had impaired ADLs, aggression, positive psychotic symptoms, cognitive impairment, delirium and falls. The use of alternative strategies such as non-pharmacological and person-centered management strategies to meet the needs of older people with these presentations should be implemented first. Staff education and support programs could improve practice and ultimately protect our older people from potential maltreatment. The use of CIs in inpatient psychiatric units should be incorporated as a quality improvement activity to monitor changes at various service provision levels. The use of CIs should be reported publicly as is already done in long-term care.

## Data Availability Statement

The data analyzed in this study is subject to the following licenses/restrictions: The data used in this study are made available to interRAI fellows for research use (not commercial use) under an existing license agreement between interRAI and the Canadian Institute for Health Information. The data in this study cannot be transmitted to third parties or made available to others. Request to access and use the data can be made directly to the Canadian Institute for Health Information. https://www.cihi.ca/en/access-data-and-reports/make-a-data-request. Requests to access these datasets should be directed to https://www.cihi.ca/en/access-data-and-reports/make-a-data-request.

## Ethics Statement

Ethics approval was obtained through the Office of Research at the University of Waterloo (ORE # 30372 and #15436). Written informed consent for participation was not required for this study in accordance with the national legislation and the institutional requirements.

## Author Contributions

GC and JPH designed the study and wrote the paper. JPH carried out the statistical analysis. TMM and YB critically reviewed the paper and contributed to writing the paper. All authors listed have made a substantial, direct and intellectual contribution to the work, and approved it for publication.

## Funding

GC was awarded with the 2018 Sir John Logan Campbell Medical Fellowship, which provided traveling expenses for this research collaboration.

## Conflict of Interest

The authors declare that the research was conducted in the absence of any commercial or financial relationships that could be construed as a potential conflict of interest.

## Publisher's Note

All claims expressed in this article are solely those of the authors and do not necessarily represent those of their affiliated organizations, or those of the publisher, the editors and the reviewers. Any product that may be evaluated in this article, or claim that may be made by its manufacturer, is not guaranteed or endorsed by the publisher.

## Footnotes

^1^Note in Ontario, an earlier version of the interRAI MH known as the Resident Assessment Instrument Mental Health (RAI-MH) is used. The interRAI MH replaced the RAI-MH as the international standard in 2007.
